# EXPLORING SECONDARY STRESSORS ON ADRD CAREGIVERS’ EXPERIENCES WITH HOME AND COMMUNITY-BASED SERVICES (HCBS)

**DOI:** 10.1093/geroni/igae098.0164

**Published:** 2024-12-31

**Authors:** Francesca Falzarano, Megan McCarthy, Hannah Mason, Annabelle Greenfield, Francesco Osso

**Affiliations:** University of Southern California, Los Angeles, California, United States; University of Colorado Firearm Injury Prevention Initiative, Denver, Colorado, United States; Weill Cornell Medicine, New York, New York, United States; University of Southern California, San Marcos, California, United States; Fordham University, New York, New York, United States

## Abstract

Family caregivers are the primary care providers for the growing population with Alzheimer’s Disease and Related Dementias (ADRD), yet they often feel unprepared and ill-equipped to carry out their role. While caregivers report high unmet needs, the low uptake of home- and community-based services (HCBS) underscores major gaps between the family care unit and the formal healthcare system. Further, little research has examined how stressors outside of direct care provision influence how caregivers perceive HCBS. Thus, using the stress process model as a theoretical framework, the current study examines the differential influences of secondary stressors on caregivers’ experiences with and attitudes toward HCBS, and its subsequent impact on psychosocial outcomes. ADRD caregivers (n=63) completed an online survey of measures assessing multiple dimensions of care-related stress. Path analyses were used to test caregivers’ perceived experiences with HCBS as mediators influencing the relationship between care-related stress and psychosocial outcomes. Results showed that those with greater family conflict reported more negative experiences with HCBS, which subsequently predicted greater levels of burden. The magnitude of the direct effect was significantly reduced and became non-significant (from β=.26, p<.05 to β=.16, p=.24), providing evidence of full mediation. Additionally, difficulty finding services partially mediated the relationship between cost-of-care and role captivity on burden, self-efficacy, and depression. Findings underscore the need for additional research focused on gaining a more holistic understanding of factors that influence how caregivers interface with the HCBS to promote advancement and continuity across the needs-to-service-use continuum.

